# Strengthening uptake of adolescent-friendly health services in Blantyre, Malawi

**DOI:** 10.4102/jphia.v17i1.1613

**Published:** 2026-04-15

**Authors:** Grace C. Sibande, Rakgadi G. Malapela

**Affiliations:** 1 Department of Health Studies, College of Human Sciences, University of South Africa, Pretoria, South Africa

**Keywords:** adolescents, adolescent-friendly health services, contraceptives, enhance, strategies, uptake

## Abstract

**Background:**

Adolescent-friendly health services (AFHSs) remain underutilised across the sub-Saharan African region. Identifying strategies is crucial to enhancing uptake and mitigating impact.

**Aim:**

To explore factors influencing the uptake of AFHSs, identify key barriers and develop strategies to enhance their utilisation.

**Setting:**

The research was conducted at four selected health facilities within Blantyre District, Malawi.

**Methods:**

An explanatory sequential mixed-methods design was employed. In the first phase, a quantitative survey was administered to a randomly selected sample of 293 adolescents using a multistage sampling approach. Data were analysed using Statistical Package for Social Sciences (SPSS) version 26, employing Chi-square tests (*p* < 0.05) to assess associations between variables and regression analysis to determine the influence of independent variables on service uptake. In the second phase, qualitative data were collected through individual interviews with six healthcare providers and three focus group discussions involving 24 adolescents. Thematic analysis was used to analyse qualitative data.

**Results:**

Findings revealed a low uptake of AFHSs at 43%. Factors influencing the low uptake included limited awareness of AFHSs, absence of designated adolescent spaces, negative provider attitudes, lack of recreational activities, frequent stock-outs of medical supplies and service preference patterns amongst adolescents. Based on these findings, nine context-specific strategies were developed to enhance access and uptake of AFHSs.

**Conclusion:**

The proposed strategies have the potential to improve the uptake of AFHSs and, consequently, increase contraceptive use amongst adolescents.

**Contribution:**

This study highlights critical barriers to the uptake of AFHSs and provides targeted strategies to address them.

## Introduction

Uptake of adolescent-friendly health services (AFHSs) is crucial for promoting the well-being and health of young populations, particularly in low-resource settings. Adolescent-friendly health services are tailored to address the specific needs of young people by addressing both their physical and mental health requirements in a manner that is accessible, acceptable, equitable, appropriate and tailored to their specific needs.^1^ These services also need to be provided with respect and in a non-judgemental manner.^2^

Though Malawi introduced AFHSs to improve contraceptive access amongst young people and create a more adolescent-centred approach, available evidence indicates that these services remain underused.^3^ Similarly, in parts of Asia, East and Sub-Saharan Africa, underutilisation of these services remains a significant challenge.^4,5^ Factors contributing to this underutilisation may include barriers related to stigma, lack of awareness, and, often, services are not adequately adapted to address the varying needs of young people. Moreover, systemic challenges such as limited resources and inadequate training for healthcare providers exacerbate the situation.^6,7^

Teenage pregnancy remains a major concern in many developing countries. It significantly impacts the health of adolescent girls, often leading to emotional and social challenges such as depression, suicidal ideation, rejection by family, unsafe abortion and leaving school prematurely.^8,9^ Malawi also faces a high teenage pregnancy rate, currently at 29%.^10^ This is concerning, especially considering that unintended pregnancies are largely preventable through contraceptive use by young people, with these methods being readily accessible at AFHSs.

Pregnancy and childbirth complications are the leading cause of death amongst 15-year to 19-year-old girls globally.^11^ Malawi’s maternal mortality rate is at 349.^12^ Unsafe abortion is the main cause of maternal deaths. At least 23% of all maternal deaths are due to complications of unsafe abortions, which also includes adolescents.^13^ In addition, providing care for incomplete abortions and managing complications like sepsis and haemorrhage places an added strain on the already scarce resources in low-income countries.^12^

The high prevalence of adolescent pregnancies and the increased cases of unsafe abortions in Malawi have raised concerns that suboptimal utilisation of AFHSs may be undermining their intended objective of enhancing access to contraceptives amongst adolescents and youths. Though previous studies have identified certain determinants of AFHSs uptake, empirical evidence on effective strategies to improve service uptake remains limited, particularly within the Malawian context.

Accordingly, this study aimed to explore factors influencing the uptake of AFHSs and identify key barriers, with the goal of informing the development of strategies to strengthen service uptake amongst adolescents in Blantyre, Malawi. The specific objectives were to determine the accessibility of AFHSs, assess adolescents’ knowledge of AFHSs and sexual and reproductive health (SRH) issues, explore factors influencing AFHS uptake and identify barriers to AFHS utilisation.

### Theoretical framework

The Health Belief Model (HBM) guided this study, providing a framework to understand adolescents’ resistance to preventive measures. Such resistance may relate to perceived susceptibility, severity, benefits and barriers. The model also considers cues to action, such as media exposure, and modifying factors, socio-psychological and structural, that may indirectly influence health behaviours.^14^

## Research methods and design

### Study design

The study employed an explanatory sequential mixed-methods design, consisting of a quantitative cross-sectional descriptive survey, followed by a qualitative explanatory phase. The quantitative phase was conducted to identify factors influencing the uptake of AFHSs, whilst the qualitative phase explored participants’ views and experiences to explain and elaborate on the quantitative findings.

### Methodology for the development of strategies

The Objectives-Goals-Strategies-Measures (OGSM) approach was applied^15^ with input from four experts in the fields of SRH and AFHSs, to guide the development of strategies. The OGSM framework helps convert objectives, goals, strategies and measures into practical and implementable action plans.

Based on the findings from phases one and two of the study, potential strategies to enhance access to AFHSs were developed.^16^ The initial strategies were reviewed and discussed with four experts in SRH and AFHSs, resulting in a consensus on nine agreed-upon strategies. The researcher and the four experts then refined these strategies before submitting them to 10 additional SRH and AFHSs experts for validation.

### Study setting

The study took place in four health facilities (referred to as health centres in Malawi) and their respective catchment areas within Blantyre District. Mdeka and Madziabango represented rural sites, whilst Ndirande and Chilomoni represented urban areas. Health centres form the backbone of primary health care, providing essential preventive, promotive, curative and rehabilitative services, including outpatient, maternal and child health, and AFHSs.

### The quantitative strand

#### Study population and sampling strategy

Phase one involved participants aged 10 years – 24 years, aligning with the World Health Organization (WHO) definition of ‘young people’, which includes both adolescents (10 years – 19 years) and youth (15 years – 24 years). This age range was selected because AFHSs are accessed by both groups. The inclusion criteria were unmarried boys and girls aged 10 years – 24 years who could communicate in either English or Chichewa. Excluded were those in the same age range but who were not residents of the selected catchment areas. The sample size was calculated using the Leslie Kish formula^17^ (Equation 1):

n=z(α2)2p(1−p)d2 [Eqn 1]

Where:

*n* = Sample size

*z* (α/2)2 = Confidence interval (1.96 for 95% confidence)

*p* = Proportion of utilisation of AFHSs in Malawi (13%).^18^

*d* = 0.05 (±5% precision). desired absolute precision (Margin of error)

Therefore, *n* = 174

A design effect of 1.5 was applied due to the use of multistage sampling.^19^

Then: 174 * 1.5 (design effect)

  *n* = 261

Multistage (cluster) sampling increases variance because observations within clusters are correlated. The design effect (Deff) accounts for this increased variance by adjusting the sample size accordingly.^19^ Though the initial sample size assumed a 100% response rate, a 10% oversampling was conducted to account for potential non-response or withdrawals, as is common in cross-sectional surveys when moderate cooperation is expected. This prevents loss of statistical power and reduces bias due to an insufficient sample size.^20^ Data were collected from 293 participants instead of the calculated 288 participants. Respondents were proportionally allocated across rural and urban sites.

### Data collection instrument

Phase one data were collected using a structured paper-based questionnaire, standardised for validity and reliability. Items were derived from the study objectives, literature and HBM constructs, and reviewed by experts for content and construct validity. The questionnaire was translated into Chichewa, back translated for consistency and pretested at the Gateway centre, resulting in minor revisions. Reliability was confirmed through internal consistency, and trained data collectors administered the tool.

### Data collection

Data collection took place from 20 September 2022 to 03 October 2022, with support from four trained research assistants. Each participant was given an information sheet to read prior to obtaining informed consent or assent. Those aged 18 years and above provided consent through a signature or fingerprint, whilst minors provided assent after their parents or guardians had obtained consent. Participants were informed of their right to withdraw at any time without penalty. Data were collected through interviewer-administered structured questionnaires by trained research assistants. This method was used because some adolescents were young or had limited literacy. Research assistants read questions aloud in the local language and recorded responses. All 288 targeted participants and five additional eligible adolescents completed the survey, yielding a 100% response rate.

### Data analysis

The outcome variable in this study was uptake of AFHSs. Data were analysed using IBM^®^ Statistical Package for Social Sciences (SPSS) statistics. Descriptive statistics, including frequencies and percentages, were used to summarise participants’ characteristics and levels of AFHSs uptake. Bivariate analyses using Chi-square tests were performed to examine associations between AFHSs uptake and selected independent variables. Variables that showed significant associations at the bivariate level were entered into a multivariable logistic regression model. A binary logistic regression model was fitted to identify independent predictors of AFHSs uptake, with unadjusted and adjusted odds ratios (AORs), 95% confidence intervals (CIs) and *p*-values reported. Multicollinearity was assessed using the variance inflation factor (VIF) and tolerance values, with no evidence detected (VIF < 10, tolerance > 0.1). Model fit was confirmed using the Hosmer–Lemeshow test (*p* > 0.05).

### Ethical considerations

Ethical clearance to conduct this study was obtained from the University of South Africa (UNISA) College of Human Science Ethics Committee (No. 67129765_CREC_CHS_2021). Ethical approval was obtained prior to the study, as research involving human participants must ensure the protection of their rights.^21^ The research proposal was reviewed and approved. Ethics approval is valid from 29 October 2021 to 29 October 2026. The research proposal was also submitted to the National Commission for Science and Technology of Malawi for ethical clearance (Ref No: NCST/RTT/2/6 PROTOCOL NO. P.08/22/663). Permission was also obtained from the Blantyre District Health Office, which oversees the four participating health facilities. Informed consent, parental consent or assent forms were reviewed with each participant, and signatures or fingerprints were collected from all respondents, including parents or guardians of participants under 18 years. Participants were assured of confidentiality, and all data were anonymised to protect their identities.

## Results

### The qualitative strand

#### Study population and sampling strategy

Phase two employed purposive sampling for both service providers and focus group discussion (FGD) participants. For individual interviews, participants included male and female service providers working at AFHSs facilities during the data collection period. Participants in the FGD met the same inclusion criteria as those in the quantitative phase.

### Data collection

Phase two data were collected in October 2022 by the researcher and four trained assistants. Two tools were used: a semi-structured interview guide for individual interviews and a discussion guide for focus groups. Both were translated into Chichewa by a language expert. Six AFHS providers from the four health facilities were individually interviewed for 45 min–60 min. Three FGDs (*N* = 24) were conducted across three randomly selected centres. Each focus group had eight people. Each discussion lasted up to 1 h. All sessions were audio-recorded with participants’ consent.

### Quantitative strand

#### Demographic profile of the respondents, *N* = 293

A total of 293 respondents participated in the survey. The majority were female (*n* = 152; 51.9%) and adolescents aged 15 years – 19 years (*n* = 143; 48.8%). Most lived with both parents (*n* = 136; 46.6%), followed by those living with their mothers only (79; 27%). The majority identified as Christian (*n* = 271; 92.5%), and most were students (*n* = 170; 58%), which aligns with the predominant age group. The second-largest group was the unemployed (*n* = 46; 15.7%).

### Accessibility of adolescent-friendly health services

The study revealed that only 43% of participants had ever accessed adolescent-friendly health services.

### Reproductive health services knowledge levels

Overall, 66.2% of respondents (*n* = 194) were aware of AFHSs, mainly informed by friends (56.7%) and nurses (13.9%). For preferred sources of SRH information, nurses (45.7%) and parents (20.5%) ranked highest. However, 61.8% had never seen facility signage indicating AFHS availability, and 65.5% had never received printed reproductive health materials (Table 1).

**TABLE 1:** Respondents’ distribution by knowledge factors (*N* = 293).^16^

Characteristics	Frequency (*n*)	%
**Heard of AFHSs**		
Yes	194	66.2
No	99	33.8
**Information source for AFHSs (*n* = 194)**		
Friends	110	56.7
Parents or guardians	16	8.2
Nurse	27	13.9
Media	16	8.2
Community leaders	22	1.5
Others	3	-
**Knowledge about services offered at AFHSs**		
Yes	155	52.9
No	114	47.1
**Received education on SRH**		
Yes	231	78.8
No	62	32.1
**Where was education obtained?**		
Community	10	4.3
Youth clubs	99	42.9
Health facility	25	10.8
Friends	3	1.3
Parents	3	1.3
School	91	39.4
**Preferred provider of SRH information**		
Parents	60	20.5
Family	16	5.5
Community	6	2.0
Grandparents	11	3.8
Friends	61	20.8
Nurse	134	45.7
**Ever seen AFHSs signpost**		
Yes	105	38.2
No	181	61.8
**Ever received SRH educational materials**		
Yes	11	34.1
No	192	65.5

AFHS, adolescent-friendly health service; SRH, sexual and reproductive health.

### Barriers to adolescent-friendly health services

Major barriers to AFHS access included shyness (86.1%) and fear of being seen at the facility (87.1%). Nonetheless, most respondents were satisfied with working days (86.7%), operating hours (93.1%), outreach services (87.6%) and facility cleanliness (92.2%). Notably, 96.9% reported that cultural and religious values were supportive of AFHS use (Table 2).

**TABLE 2:** Possible barriers to the utilisation of adolescent-friendly health services (*N* = 293).^16^

Characteristics	Frequency (*n*)	%
**Shyness to attend AFHSs (*n* = 266)**		
Yes	37	13.9
No	229	86.1
**Feared to be seen at AFHSs (*n* = 264)**		
Yes	34	12.9
No	230	87.1
**Fine with AFHSs working days (*n* = 166)**		
Yes	144	86.7
No	22	13.3
**Fine with operation times (*n* = 159)**		
Yes	148	93.1
No	11	6.9
**Fine with AFHS outreach days (*n* = 153)**		
Yes	134	87.6
No	19	12.4
**Cleanliness of environment (*n* = 141)**		
Yes	130	92.2
No	11	7.8
**Requires the company to access AFHSs (*n* = 250)**		
Yes	30	12.0
No	220	88.0
**Has no problem with familiar faces at an AFHSs facility (*n* = 260)**		
Yes	229	88.1
No	31	11.9
**Cultural support for use of AFHSs (*n* = 289)**		
Yes	280	96.9
No	9	3.1
**Religious support for use of AFHSs (*n* = 289)**		
Yes	280	96.9
No	9	3.1

AFHS, adolescent-friendly health service.

### Associations

Cross-tabulations and Pearson’s Chi-square tests (α = 0.05, 95% confidence interval [CI]) identified key factors associated with AFHS uptake in Blantyre. As shown in Table 3, significant variables (*p* < 0.05) included gender, age, AFHS awareness, prior SRH education, presence of signposts and encouragement or discouragement to use services. Additional factors such as respectful provider attitudes, embarrassment and fear of being seen were also significant and included in subsequent analyses.

**TABLE 3:** Cross-tabulations and Pearson’s Chi-square results.^16^

Characteristics	Yes (*n*)	No (*n*)	*n*	*χ* ^2^	*p*-value
Gender	-	-	-	6.07	0.048
Male	52	89	141	-	-
Female	73	75	148	-	-
Age (years)	-	-	-	16.12	≤ 0.003
10–14	20	53	72	-	-
15–19	59	82	143	-	-
20–24	46	32	78	-	-
Aware of the services provided by AFHSs	-	-	-	115.69	≤ 0.001
Yes	114	40	154	-	-
No	9	104	114	-	-
Exposure to SRH information	-	-	-	16.57	≤ 0.001
Yes	111	228	229	-	-
No	14	45	59	-	-
Noticed a sign indicating the presence of AFHSs	-	-	-	49.22	≤ 0.001
Yes	71	31	102	-	-
No	51	130	181	-	-
Provided with SRH informational materials	-	-	-	32.99	≤ 0.001
Yes	64	35	99	-	-
No	60	131	191	-	-
Received encouragement to use AFHSs	-	-	-	420.59	≤ 0.001
Yes	117	42	159	-	-
No	8	119	127	-	-
Discouraged from utilising AFHSs	-	-	-	26.45	≤ 0.001
Yes	55	31	76	-	-
No	66	129	195	-	-
Handled with respect by the service provider	-	-	-	8.211	0.016
Yes	105	11	116	-	-
No	4	3	7	-	-
Religious backing for AFHSs use (*n* = 289)	-	-	-	17.07	≤ 0.001
Yes	105	12	117	-	-
No	5	2	7	-	-
Was reluctant to access AFHSs due to shyness	-	-	-	6.80	0.033
Yes	10	27	37	-	-
No	113	114	228	-	-
Worried about being noticed whilst at AFHSs	-	-	-	6.75	0.034
Yes	9	25	34	-	-
No	114	115	229	-	-

AFHS, adolescent-friendly health service; SRH, sexual and reproductive health.

### Predictors of adolescent-friendly health services uptake

Multivariable logistic regression identified several key predictors of AFHS utilisation (Table 4). Adolescents’ demographic characteristics influenced uptake: males (Adjusted odds ratio [AOR] = 0.61, 95% CI: 0.37–0.98, *p* = 0.039) and those aged below 20 years (AOR = 0.19, 95% CI: 0.06–0.59, *p* = 0.001) were less likely to use AFHSs compared to females and older youths, respectively.

**TABLE 4:** Predictors of uptake of adolescent-friendly health services.^16^

Characteristics	Unadjusted OR	95% CI	AOR	95% CI	*p*-value
**Gender**					
Male	1.00	-	1.00	-	1.000
Female	0.60	0.37, 0.96	0.61	0.37, 0.98	0.039*
**Age (years)**					
10–14_RC_	1.00	-	1.00	-	1.000
15–19	0.53	0.29, 0.99	0.65	0.32, 1.35	0.245
20–24	0.27	0.13, 0.55	0.19	0.06, 0.59	0.001*
**Aware of the services provided by AFHSs**					
No_RC_	1.00	-	1.00	-	1.000
Yes	33.54	12.05, 93.35	28.61	10.99, 74.45	0.000*
**Exposure to SRH information**					
No_RC_	1.00	-	1.00	-	1.000
Yes	3.09	1.59, 6.02	2.72	1.39, 5.32	0.002*
**Noticed a sign indicating presence of AFHSs**					
No_RC_	1.00	-	1.00	-	1.000
Yes	6.08	3.39, 10.92	5.58	3.14, 9.91	0.000*
**Provided with SRH informational materials**					
No_RC_	1.00	-	1.00	-	1.000
Yes	3.92	2.28, 6.74	3.74	2.17, 6.44	0.000*
**Received encouragement to use AFHSs**					
No_RC_	100	-	1.00	-	1.000
Yes	54.78	15.97, 187.92	52.26	15.41, 177.19	0.000*
**Discouraged from utilising AFHSs**					
No_RC_	1.00	-	1.00	-	1.000
Yes	3.53	2.03, 6.14	3.41	1.94, 6.00	0.000*
**Handled with respect by the service provider**					
No_RC_	1.00	-	1.00	-	1.000
Yes	7.22	1.34, 38.69	6.34	1.28, 31.43	0.009*
**Demonstrated to by the provider**					
No_RC_	1.00	-	1.00	-	1.000
Yes	3.53	0.60, 20.70	3.82	0.69, 20.94	0.045*
**Was reluctant to access AFHS due to shyness**					
No_RC_	1.00	-	1.00	-	1.000
Yes	0.37	0.17, 0.81	0.37	0.16, 0.83	0.012*
**Fear of being seen at AFHS**					
No_RC_	1.00	-	1.00	-	1.000
Yes	0.36	0.15, 0.81	0.38	0.16, 0.88	0.020*

Note: The analysis employed a logistic regression model with a sample size of 293 and a significance level set at *p* = 0.05.

OR, odds ratio; AOR, adjusted odds ratio; CI, confidence interval; AFHS, adolescent-friendly health service; SRH, sexual and reproductive health; AFHS, adolescent-friendly health services; _RC_, Indicates the reference category.

* , Signifies a statistically significant category.

Knowledge and awareness-related factors strongly increased utilisation. Adolescents who were aware of AFHS services (AOR = 28.61, 95% CI: 10.99–74.45, *p* < 0.001), exposed to SRH information (AOR = 2.72, 95% CI: 1.39–5.32, *p* = 0.002), noticed service signs (AOR = 5.58, 95% CI: 3.14–9.91, *p* < 0.001) or received SRH informational materials (AOR = 3.74, 95% CI: 2.17–6.44, *p* < 0.001) were significantly more likely to utilise the services. Encouragement from others had the strongest effect (AOR = 52.26, 95% CI: 15.41–177.19, *p* < 0.001).

Attitudinal and facility-related factors also played a role. Adolescents who were reluctant due to shyness (AOR = 0.37, 95% CI: 0.16–0.83, *p* = 0.012) or feared being seen (AOR = 0.38, 95% CI: 0.16–0.88, *p* = 0.020) were less likely to use AFHSs, whereas respectful treatment by providers increased uptake (AOR = 6.34, 95% CI: 1.28–31.43, *p* = 0.009).

### The qualitative strand

Data from individual interviews and FGDs were analysed using Braun and Clarke’s thematic analysis approach, following their six-step framework as outlined in their 2006 publication.^16^ Two researchers independently coded transcripts, compared their interpretations and refined themes through a consensus process. Reflexivity was maintained through peer debriefing and the documentation of analytic decisions, which enhanced the trustworthiness of the research.

The demographic profile of the youth participants (*N* = 24) included an equal number of males and females in each focus group. Each group consisted of eight members – one male and one female from each of the following age groups: 10 years – 12 years, 13 years – 16 years, 17 years – 20 years and 21 years – 24 years. All youth participants were unmarried.

For the individual interviews, participants were all aged above 24 years. Amongst them, five were female and one male, with one participant being married. All were identified as Christians. Two participants had tertiary education and were nurses, whilst two had completed secondary education. Only one participant had received formal training in delivering AFHSs; the others had been given only on-the-job orientation. All had worked at a AFHS facility for more than a year, with one having over 7 years of experience.

### Key themes and sub-themes

Thematic analysis of verbatim transcripts from both individual interviews and FGDs yielded six key themes. A summary of these themes, along with their corresponding sub-themes, is presented in Table 5.

**TABLE 5:** Themes and Sub-themes.^16^

Themes	Sub-themes
1: Awareness of SRH and AFHSs	-
2: Availability and accessibility of AFHSs	-
3: Factors encouraging uptake of AFHSs	-
4: Challenges hindering access to AFHSs	4.1: Frequent shortage of contraceptives and medical supplies 4.2: Adolescent prioritisation of entertainment and sports over health services 4.3: Absence of a designated space for AFHSs 4.4: Long distance to the health facility 4.5: Misconceptions about sexual and reproductive health issues and adolescent-friendly health services
5: Adolescent preferences	-
6: Attitudes of service providers	-

AFHS, adolescent-friendly health service; SRH, sexual and reproductive health.

#### Theme 1: Awareness of sexual and reproductive health and adolescent-friendly health services

Focus group participants acknowledged that whilst some adolescents were informed about SRH, others lacked this knowledge, which may have discouraged them from using AFHSs. Similarly, service providers noted that awareness of SRH and the availability of AFHSs were not universal amongst adolescents in the catchment areas. Some participants directly stated:

‘Yeah … adolescents are knowledgeable about sexual reproductive health issues through organisations such as MANASO, but I can say that not all the adolescents are knowledgeable.’ (Service provider 1, Madziabango, P1)

As to where the adolescents got the information, the following answer was received from one FGD:

‘Ahem … mostly we get the information from the hospital. Yes … parents do provide us with information, but it is not rich. We get more information from the health workers.’ (FGD 2, Mdeka, P6)

#### Theme 2: Availability and accessibility of adolescent-friendly health services

The overall perception was that the utilisation of AFHSs was low.^16^ Service providers reported that on the best days, attendance could reach up to 40 youths, but in most cases, turnout was minimal, particularly when no entertainment or sporting events were offered at the facility. One participant explained:

‘Yes … the services are accessible to some adolescents but not accessible to others due to distance and stockouts of supplies, including contraceptives.’ (Service provider 3, Lirangwe, P3)

A recurring point raised regarding the accessibility of AFHSs was that sports and entertainment activities played a key role in drawing adolescents to AFHS. As some participants noted:

‘Eeeh … Sometimes, youth bonanzas are organised by NGOs, such as Jhpiego, which provides reproductive health education to youths aged 15 [*years*] to 24 [*years*] in the communities. This somehow contributes to increasing accessibility to YFHS.’ (Service provider 1, Madziabango, P1)

#### Theme 3: Factors encouraging uptake of adolescent-friendly health services

Participants shared various factors that encouraged their continued use of AFHSs. These included the valuable health information they received, particularly on preventing sexually transmitted infections and unplanned pregnancies. The following quote illustrates what motivated adolescents to access AFHSs:

‘Yeah … We learn different things such as how to use condoms correctly … use of emergency contraceptive pills, we also learn about HIV and AIDs, how we can prevent contracting it and also the importance of HIV testing.’ (FGD1, Madziabango, P1)

#### Theme 4: Challenges hindering access to adolescent-friendly health services

Participants indicated that multiple obstacles hindered the utilisation of AFHS.

**Sub-theme 4.1: Frequent shortage of contraceptives and medical supplies**^16^: Adolescent participants noted that apart from condoms, most contraceptive methods were not consistently available at health facilities which discouraged some from seeking contraceptive services at AFHS. Service providers across all the health facilities involved in the study confirmed this, acknowledging frequent shortages of supplies, including contraceptives:

‘Yeah … also … lack of contraceptive methods that we want. Sometimes we want the injection method, but we are told that it is not available two or three times. Due to that, we get discouraged and we stop coming to AFHS and go elsewhere to be assisted.’ (FGD3, Ndirande, P5)

**Sub-theme 4.2: Adolescent prioritisation of entertainment and sports over health services:** Adolescent participants explained that they valued sporting and recreational activities more.^16^ The adolescents were of the view that the availability of sporting activities at AFHSs could influence adolescents to patronise the services. These sentiments were also shared by service providers.

One notable quote was:

‘Okay … an adolescent is like a child. He will go where there are games as opposed to where there is health education only. As such, we need to have balls and other resources such as game boards, playing cards. These should always be available at the health facility and will attract us as adolescents and more adolescents to attend AFHSs.’ (FGD2, Mdeka, P5)

**Sub-theme 4.3: Absence of designated space for adolescent-friendly health services and adolescent-friendly health services:** Participants across all the sites reported that there were no designated rooms or specific areas allocated for AFHSs. Instead, these services were often conducted in spaces intended for other purposes, such as antenatal clinics, which affected the level of privacy.^16^ As one service provider explained:

‘Yeah … another reason is that we have no specific place for AFHSs, and we conduct our services in an open space … some adolescents feel shy to come, fearing people who know them will see them and maybe report them to their parents, hence they don’t come.’ (FGD2, Mdeka, P5)

**Sub-theme 4.4: Long distance to the health facility:** Participants identified long travel distances as a barrier to accessing AFHSs, particularly for adolescents living in large and hilly catchment areas:

‘Eeeh … This health facility has a big catchment area, which makes it difficult for adolescents to walk long distances …’ (Service provider 3, Chilomoni, P5)

**Sub-theme 4.5: Misconceptions about sexual and reproductive health issues and adolescent-friendly health services:** The study revealed that widespread misconceptions about contraceptive use amongst parents and the broader community were discouraging adolescents from accessing AFHSs. Some of the participants expressed this through the following statements:

‘Ummh … the woman will not be sexually active … we also hear the women who use contraceptives are not sweet’ [*laughs*]. It is not good indeed because some people also say contraceptives reduce male libido.’ (FGD1, Madziabango, P4)

‘Yes … many parents also refuse because they believe it will lead to barrenness among girls.’ (Service provider 4, Madziabango, P2)

#### Theme 5: Adolescent preferences

Adolescent participants from all the health facilities expressed specific preferences, particularly regarding timing. Some favoured attending AFHSs activities on weekends, as weekdays were often occupied with school or other commitments:

‘Sunday afternoon is perfect as it does not burden anyone.’ (FGD2, Lirangwe, P3)

‘Saturday is fine, and it has to be at the hospital and not at school or in the village.’ (FGD3, Ndirande, P2)

Participants stated that they preferred to have a dedicated space for AFHSs. Some of them shared the following views:

‘Ummh … Since we do not have a special room for AFHSs, when we come during weekdays, there is a lack of privacy and confidentiality in the provision of services because they are offered together with the adults, where you can bump into your father whilst collecting condoms.’ (FGD1, Madziabango, P2)

‘Better fellow youths than older people.’ (FGD1, Madziabango, All participants)

‘As for me, Ummh … I prefer youthful health workers because I can easily open up with a fellow youth.’ (FGD2, Lirangwe, P5)

When it came to the gender of service providers, most participants favoured having both male and female providers available at AFHSs.^16^ Nonetheless, a significant number of adolescents preferred providers of the same gender, explaining that they felt more comfortable and found it easier to communicate openly with them. Some participants noted:

‘As for me … I would rather be seen by a fellow man because I will be freer. Of course, not that a female provider cannot see me, but I think it is better to have both genders for the sake of those who feel uncomfortable with the opposite gender.’ (FGD2, Lirangwe, P2)

‘Ummh … If we were to choose, I would choose a fellow female provider. I would not feel shy with such a provider than a male provider.’ (FGD3, Ndirande, P5)

#### Theme 6: Attitudes of service providers

Participants in the study expressed overall satisfaction with the attitudes of the service providers. They described them as approachable, friendly, welcoming, non-judgemental and respectful in their interactions with adolescents. Service providers affirmed these perceptions, stating that their openness and supportive approach helped adolescents feel more comfortable and willing to share. Some participants commented:

‘Eeeh … we do not have any issues with them. They welcome us well and they listen to our problems.’ (FGD2, Lirangwe, P5).

‘Yeah … they are friendly to us too.’ (FGD2, Lirangwe, P2)

‘Yes … they also treat us well when we come to the hospital with other problems on days that are not for AFHSs.’ (FGD2, Lirangwe, P6)

### Development of strategies for improving uptake of adolescent-friendly health services

**FIGURE 1:**
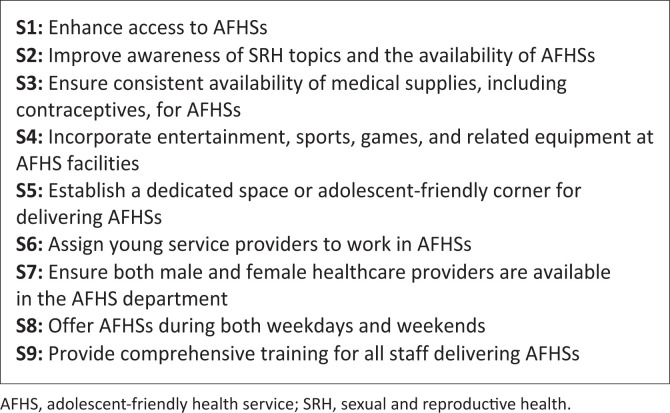
Illustration of proposed strategies to improve uptake of adolescent-friendly health services.

The nine strategies were informed in phase three by both quantitative and qualitative data: Daily services, youthful and gender-diverse providers, dedicated spaces and flexible hours, addressed accessibility and comfort, supported by survey results and the expressed preferences of adolescents. Awareness campaigns, recreational activities, outreach services and parent engagement highlighted gaps in knowledge, engagement and family support, underscoring the need for an evidence-based, youth-centred approach to improving AFHSs uptake. (Refer to Figure 1 for the strategies.)

The developed strategies incorporated novel, context-specific elements that extend beyond existing national guidelines. These included the integration of entertainment and recreational activities within AFHSs settings (S4), enhanced awareness initiatives to inform adolescents about available services (S2) and the provision of services daily rather than on select days (S1). Participants further emphasised the importance of dedicated service spaces (S5), engagement of youthful providers (S6) and the presence of both male and female staff to accommodate gender preferences (S7).

## Discussion

The study highlights that knowledge of SRH and AFHSs has a significant influence on service uptake. About 66.2% of respondents were aware of AFHSs and the services they offer, whilst 78.2% had received SRH information. Service providers confirmed that some adolescents were knowledgeable in these areas. However, a contrasting study in Ethiopia reveals low SRH awareness as a barrier to accessing AFHSs.^22^ In this study, nurses were the preferred source of SRH information (45.7%), followed by parents (20.5%). However, qualitative data revealed that adolescents considered parental information to be superficial. Like earlier studies, other trusted sources of SRH information included family,^23^ peers,^24^ media and static advertisements.^25^ However, printed SRH materials were lacking as 65.5% of respondents had never received any, which negatively impacted service uptake, emphasising the need for such materials at AFHS departments.

Visibility of AFHSs also mattered. Over half of the respondents (61.8%) reported never seeing a signpost indicating AFHS availability, which may contribute to low service uptake. Supporting findings from Ethiopia showed that the lack of signposts hindered uptake,^26^ suggesting the necessity for the availability of AFHSs’ signposts.

Access remained a challenge, with only 43% of youths having used AFHSs. Qualitative data revealed barriers such as a lack of private spaces for AFHSs, long travel distances, limited awareness, youth disinterest due to undervaluing the services and parental disapproval stemming from fears of encouraging sexual activity. Notably, only 30% of respondents reported ever being discouraged from attending, which ideally should have meant higher uptake.

Findings from other regions echoed these challenges. For instance, adolescent AFHS use was 26.3% in a study in Southeast Ethiopia^27^ and even lower in a study conducted in Nepal^4^ (14.8%) and in Ghana (42%).^28^ However, a study in Northwest Ethiopia reported a higher rate, with 61.5% of youth using AFHSs within the previous year.^29^

This highlights the need to implement the identified strategies to improve the uptake of these services.

Whilst regional reviews highlight barriers such as limited awareness, unfriendly providers and inconvenient hours, the current data identify new demand-driven elements, including entertainment, recreation and dedicated youth service spaces that may enhance acceptability and engagement. This is consistent with evidence that service design and youth-centredness drive utilisation of AFHSs in sub-Saharan Africa.^30^ The researchers suggest partnerships with local organisations and budgeting by facility managers to provide sports equipment as a practical solution.

Adolescent participants were generally satisfied with the provider’s attitudes. They described providers as friendly, respectful and non-judgemental, which encouraged continued use of their services. Nearly all (94.4%) of those who accessed AFHSs felt treated with respect, whilst only 3% described providers as harsh. These findings align with a study in Sweden and a narrative review where respectful treatment was linked to higher service uptake.^31,32^ This underscores the importance of deploying well-trained, adolescent-friendly staff.

Consistent with other studies, this research also identified key deterrents, such as privacy concerns resulting from a lack of designated spaces for AFHSs, which hindered access, especially when services were delivered in shared spaces.^33^ This calls for the provision of designated spaces for adolescents, such as adolescent corners or setting apart rooms to be used for the provision of AFHSs.

Misconceptions about contraceptives amongst adolescents, parents and communities also hindered AFHS uptake. This aligns with a study conducted in Zambia, which found that the fear of infertility contributed to low uptake of contraceptives.^34^ This calls for community sensitisations, where such misconceptions can be dispelled.

Distance was another barrier, especially in large or hilly catchment areas, which limited mobility and discouraged adolescent attendance. Previous studies similarly found geographic inaccessibility to be a major obstacle,^35^ highlighting the need for outreach strategies.

Regarding service availability, some adolescents preferred weekend access due to school and other weekday commitments. However, some suggested that weekday availability would enable the timely treatment of urgent issues, such as sexually transmitted infections (STIs). Unlike earlier studies that cited inconvenient hours and long waits as barriers, participants in this study did not view weekend services as problematic.

Gender norms, family communication and community expectations shape adolescents’ willingness to seek care and their preferences for service providers. Participants expressed greater comfort with youthful and same-gender providers, noting that these facilitated open discussion of sensitive topics; 63.8% preferred providers closer to their age. The preference for youthful and gender-diverse staff aligns with evidence that provider age, gender and perceived judgement influence perceptions of confidentiality and respect. Moreover, youth-led outreach provides a culturally acceptable avenue for engaging adolescents, consistent with regional evidence that integrating service improvements with community-level norm interventions enhances impact.^36^

### Study strengths and limitations

The study’s findings may have limited generalizability beyond the study context. Nonetheless, the findings provide valuable insights into strategies that could enhance the uptake of AFHSs, offering useful guidance for policymakers aiming to improve access and uptake of these services. The use of self-reported data may have introduced recall and social desirability bias, given the sensitive nature of SRH questions. Additionally, some variables exhibited wide confidence intervals (e.g. encouragement AOR = 52.26), likely due to small subgroup sizes and potential model overfitting, and should therefore be interpreted with caution.

### Implications

The findings from this study highlight the importance of targeting both male and female adolescents in efforts to promote the uptake of AFHSs. Whilst girls are often more directly affected by the consequences of unintended pregnancies, it is equally important for boys to be well-informed about reproductive health to support prevention efforts. Greater attention should also be given to younger adolescents, who are at a higher risk of experiencing complications from early childbearing.

Given that most adolescents are still in school, there is a need for policies that integrate comprehensive SRH and AFHSs information into both primary and secondary education. Additionally, establishing youth clubs in remote areas where access to government health facilities is limited could help bring services closer to the community. These clubs could serve as points for AFHS delivery, with trained service providers visiting at least once a month to offer support and care.

There is also a clear need to expand SRH education and awareness efforts across multiple platforms, including schools, youth clubs, outpatient departments of health facilities and community settings. Mass media channels, such as radio and television, can be powerful tools in spreading SRH messages, especially when supported by the Ministry of Health in collaboration with Non-Governmental Organizations (NGOs) that focus on adolescent health.

Furthermore, the study emphasises the importance of deploying trained, adolescent-friendly service providers in AFHSs. Such professionals are better equipped to offer respectful, supportive and non-judgemental care, which in turn encourages greater adolescent engagement with these services.

## Conclusion

The study revealed that the uptake of AFHSs in Blantyre remained low, at 43%. Analysis identified six key factors influencing access: awareness of AFHSs, service providers’ attitudes, frequent shortages of medical supplies, absence of entertainment and sports activities with supporting equipment, lack of designated spaces for service delivery, adolescents’ preferences regarding service days and the age and gender of providers. Addressing these factors could significantly improve service utilisation. The findings call for policies that strengthen adolescent-friendly health services through a gender-inclusive, age-sensitive approach and integrating comprehensive SRH education into primary and secondary schools. Policies should also prioritise the training and deployment of adolescent-friendly service providers.

By identifying nine targeted strategies to enhance AFHSs uptake, the study addressed a critical knowledge gap. Further research is recommended to assess the friendliness of AFHSs across Malawi, as well as to evaluate the effectiveness of the proposed strategies in improving service uptake.
